# T^−786^→C polymorphism of the endothelial nitric oxide synthase gene is associated with insulin resistance in patients with ischemic or non ischemic cardiomyopathy

**DOI:** 10.1186/1471-2350-13-92

**Published:** 2012-10-02

**Authors:** Cecilia Vecoli, Maria Grazia Andreassi, Riccardo Liga, Maria Giovanna Colombo, Michele Coceani, Clara Carpeggiani, Antonio L’Abbate, Danilo Neglia

**Affiliations:** 1 CNR, Institute of Clinical Physiology, Pisa, Italy; 2 Scuola Superiore Sant’Anna, Institute of Life Sciences, Pisa, Italy; 3 Fondazione G. Monasterio CNR - Regione Toscana, Pisa, Italy

**Keywords:** eNOS polymorphism, Insulin resistance, Heart failure

## Abstract

**Background:**

Insulin resistance (IR) and endothelial dysfunction are frequently associated in cardiac disease. The T^−786^→C variant in the promoter region of the endothelial nitric oxide synthase (eNOS) gene has been associated with IR in both non-diabetic and diabetic subjects. Aim of the study was to assess the reciprocal relationships between T^−786^→C eNOS polymorphism and IR in ischemic and non-ischemic cardiomyopathy.

**Method:**

A group of 132 patients (108 males, median age 65 years) with global left ventricular (LV) dysfunction secondary to ischemic or non-ischemic heart disease was enrolled. Genotyping of T^−786^→C eNOS gene promoter, fasting glucose, insulin, and insulin resistance (defined as HOMA-IR index > 2.5) were determined in all patients.

**Results:**

Genotyping analysis yielded 37 patients homozygous for the T allele (TT), 70 heterozygotes (TC) and 25 homozygous for C (CC). Patients with CC genotype had significantly higher systemic arterial pressure, blood glucose, plasma insulin and HOMA index levels than TT. At multivariate logistic analysis, the history of hypertension and the genotype were the only predictors of IR. In particular, CC genotype increased the risk of IR (CI% 1.4-15.0, p < 0.01) 4.5-fold. The only parameter independently associated with the extent of LV dysfunction and the presence of heart failure (HF) was the HOMA index (2.4 CI% 1.1-5.6, p < 0.04).

**Conclusions:**

T^−786^→C eNOS polymorphism was the major independent determinant of IR in a population of patients with ischemic and non-ischemic cardiomyopathy. The results suggest that a condition of primitive eNOS lower expression can predispose to an impairment of glucose homeostasis, which in turn is able to affect the severity of heart disease.

## Background

Insulin resistance (IR) is a common condition in patients with both ischemic and non-ischemic cardiomyopathy. It may be secondary to heart failure (HF) syndrome but also contributes to the disease [1-3]. As IR represents a potentially reversible alteration, its early identification may have a crucial role in HF prevention and treatment [4,5].

Insulin resistance has often been linked to endothelial dysfunction, defined as paradoxical or inadequate endothelial-mediated vasodilation [6-8]. Specifically, IR has been associated with the decreased synthesis and/or release of nitric oxide (NO), as occurs in many clinical conditions including HF, or with its exaggerated consumption due to high tissue levels of reactive oxygen and nitrogen species in a condition of altered glucose and lipid metabolism [7]. Alternatively, genetically determined deficiency of endothelial-derived NO could predispose to IR. In an experimental study, Duplain et al. showed that knockout mice for the endothelial NO synthase (eNOS) gene were insulin-resistant and hypertensive, suggesting that eNOS is important not only for cardiovascular control but also for glucose homeostasis [9]. In humans, a thymidine-to-cytosine (T to C) transition mutation (T^−786^→C) in the promoter region of the gene has been associated with reduced eNOS expression [10] and has been linked to IR both in non-diabetic subjects and Type 2 diabetic patients [11,12].

Based on these premises, we hypothesized that genetic eNOS impairment could be an independent determinant of IR in patients with HF. Accordingly, in the present study we tested the reciprocal relationships between T^−786^→C eNOS polymorphism and IR in patients with ischemic and non-ischemic cardiomyopathy fully characterized for cardiac function and other cardiovascular risk factors.

## Materials and methods

### Study population

The study population consisted of 132 patients (108 males; age 65.5 ± 10.4 years) with mild to severe left ventricle (LV) systolic dysfunction with or without HF (NYHA Class I-III). Sixty-six patients had ischemic, while 66 patients had non-ischemic LV dysfunction (angiographically normal coronary arteries). The population was selected among 498 consecutive patients referred for elective coronary angiography at CNR Institute of Clinical Physiology from 2007 to 2010 and enrolled in the GENOCOR ( Genetic Mapping for Assessment of Cardiovascular Risk) study (FIRB 2005) aimed to assess genetic determinants of coronary artery disease. Selection criteria included: 1.reduced LV ejection fraction (EF) (< 50%) by the biplane area-length method on rest echocardiography; 2. optimal medical treatment with no changes in medication during the last 3 months; 3. no evidence of recent myocardial infarction or unstable angina (within the last 6 months); 4. absence of acute or unstable HF; 5. no history of diabetes; 6. no significant concomitant disease such as infections, malignancies or connective tissue disease; 7. informed written consent. A complete clinical history was collected from all patients. The following cardiovascular risk factors were recorded in every patient: age, sex, smoking habit (current smokers or ex-smokers -- within the last year), hypercholesterolemia (total cholesterol level ≥ 200 mg/dl, low-density lipoprotein [LDL] cholesterol level ≥ 100 mg/dl or treatment with lipid-lowering agents), history of arterial hypertension (arterial blood pressure ≥ 140 mmHg for systolic or ≥ 90 mmHg for diastolic, or use of anti-hypertensive medications), and obesity (body mass index ≥ 30 kg/m^2^). Severe LV dysfunction with heart failure (LVEF < 40% and NYHA Class II-III) was present in 35 patients.

Ninety-eight healthy volunteers (46 males; age 45.1 ± 9.5 years), members of the medical and technical staff of our Institution, negative for clinical cardiovascular risk factors, were used as control group in order to compare the frequency of T^−786^→C genotypes in the general population relative to patients.

The Local Ethics Committee (Comitato Etico Sperimentazione Farmaco - Azienda Ospedaliera Universitaria Pisana, Italy) approved the study and all patients gave their written informed consent. The research was conducted according to the principle of the Declaration of Helsinki

### Biohumoral characterization

Fasting plasma glucose, fasting serum insulin concentrations and homeostasis model assessment [HOMA] score were measured in all patients. Blood samples were obtained from each subject after a 12-h overnight fast by evacuation from an antecubital vein into vacutainer tubes. Fasting plasma glucose concentration was measured by a glucose oxidase method, and concentrations of total cholesterol and triglycerides were measured by enzymatic procedures using an autoanalyzer (UniCel DxC 600, Beckman Coulter). Fasting insulin concentration was measured by immunoassay (Architect i1000 sr, Ilex Medical). Insulin resistance was defined as Homeostasis Model Assessment of IR (HOMA-IR) > 2.5. The HOMA-IR was calculated from the formula: HOMA-IR = fasting serum insulin (μU/ml) x fasting plasma glucose (mmol/l)/22.5 [13].

### Analysis of T^−786^→C polymorphism in the 5’- flanking region of the eNOS gene

The presence of the T-C conversion at nucleotide position 786 in the 5’-flanking region of the eNOS gene was determined by PCR amplification with the primers 5’- ATGCTCCCACCAGGGCATCA-3’ (sense) and 5’-GTCCTTGAGTCTGACATTAGGG- 3’ (antisense), as previously reported [14]. The 236-bp PCR fragments were digested with NgOAIV restriction enzyme for 16 h at 37 °C. The wild-type allele (T) has no *NgOAIV* cleavage site, whereas the PCR product is cleaved into two fragments of 203 and 33 bp in the presence of the C^-786^ allele.

### Statistical analysis

Statistical analyses of the data were conducted with the Statview statistical package, version 5.0.1 (Abacus Concepts, Berkeley, Calif., USA). Data are expressed as mean ± SD. Because of the skewness of the distributions of biochemical values, analyses have been performed using the logarithmic transformation of data. Differences between the means of two continuous variables were evaluated by Student's *t*-test. Differences in non-continuous variables and genotype distribution were tested by χ^2^ analysis. The data for three or more independent groups were analyzed by analysis of variance, and significant differences among pairs of means were tested by Bonferroni’s test. Regression analysis with Pearson’s test was also used to evaluate the relationship between the continuous variables. Unconditional logistic regression was used to calculate odds ratio (ORs) and 95% CIs. The ORs were also adjusted for other risk factors. A p-value < 0.05 was considered significant.

Assuming a mean value of HOMA of 2.0 and standard deviation of 2.5, a study with a sample size of 130 patients would be needed to detect a 15 % difference or more in HOMA-IR between the heterozygous and the TT homozygous patients for T−786→C variant, with a power of β =80% by means of a two-sided t-test with α = 5%.

## Results

### Genotype analysis

**Figure 1:**
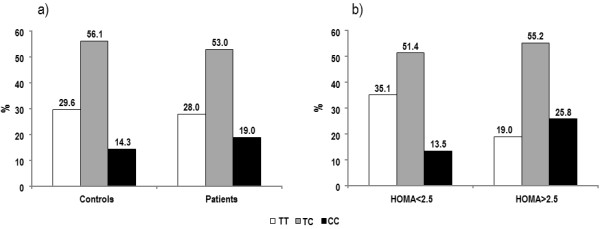
**Genotype frequencies for the polymorphism of eNOS gene promoter T**^−**786**^→**C a) in healthy subjects and patients with systolic LV dysfunction and b) in patients with HOMA**-**IR index<2**.**5 or with HOMA**-**IR>2**.**5.**

Genotyping analysis in the patient population yielded 37 patients homozygous for the T^−786^ allele (TT), 70 heterozygotes (TC) and 25 homozygous for C^-786^ (CC). The genotype distribution of the T^−786^→C polymorphism in patients was not significantly different from that observed in the control group (Figure 1a) and was comparable with that previously observed in Caucasian subjects [14]. Nevertheless, when patients were stratified by HOMA-IR index (HOMA-IR index > or < 2.5), the allelic distribution was significantly different. The frequency of CC homozygous patients was significantly higher and that of TT homozygous patients was significantly lower in patients with HOMA-IR index > 2.5 as compared to patients with HOMA-IR index < 2.5 (25.8% vs 13.5%, p < 0.05 and 19.8 vs 35.1, p <0.05) (Figure 1b).

### Clinical and biochemical evaluation

**Figure 2:**
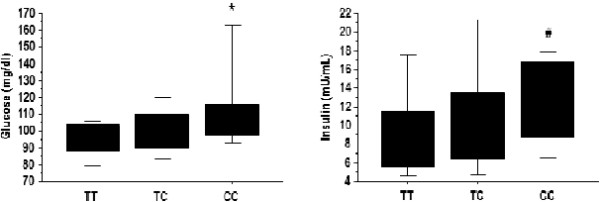
**Effects of the eNOS gene promoter T**^−**786**^→**C polymorphism on glucose and insulin levels in patients with systolic LV dysfunction.** *p=0.005 CC vs TT; #p<0.05 CC vs TT.

The demographic and clinical characteristics of the patients population are shown in Table 1. Stratifying the population according to eNOS T^−786→^C polymorphism, the three groups of patients did not differ as to age, sex, smoking, family history of coronary artery disease (CAD), obesity and systemic lipid metabolism. The three groups also did not differ as to etiology and severity of LV dysfunction or presence of overt HF. Conversely, significant differences were found in arterial hypertension and in glucose homeostasis. Indeed, a history of arterial hypertension was more frequent in CC than in TT patients. Blood glucose and insulin levels were significantly different within the three groups with an increasing trend from TT to CC patients (Figure 2). As a consequence, CC patients had higher values of HOMA-IR index compared with TC and TT patients (3.9 ± 2.8 CC vs 2.4 ±2.8 TC vs 2.2 ± 1.3 TT) and a greater prevalence of subjects with HOMA-IR index > 2.5 (60% compared with 30% in the TT group, p = 0.008).

**Table 1:** Demographic and clinical characteristics of study population

	**Whole Population n **=**132**	**TT n**=**37**	**TC n**=**70**	**CC n**=**25**	**p**-**value TT vs CC**
**Mean age **(**mean** ± **SD**)	65.4±10.4	64.3 ± 12.1	65.5 ± 10.0	67.1 ± 9.0	ns
**Gender **(**male**), **n** (%)	108 (81.8)	32 (86.5)	57 (81.4)	19 (82.6)	ns
**Smoking habit**, **n **(%)	77 (58.3)	19 (51.4)	46 (45.7)	10 (43.5)	ns
**Hypercholesterolemia n **(%)	73 (55.3)	14 (37.8)	46 (65.7)	11 (47.8)	ns
**History of Hypertension**, **n **(%)	45 (34.1)	9 (24.3)	25 (35.7)	9 (39.1)	< 0.05
**CAD Familiarity**, **n **(%)	56 (42.4)	19 (51.4)	46 (65.7)	10 (43.5)	ns
**Obesity**, **n **(%)	20 (15.2)	9 (2.4)	9 (1.3)	2 (0.9)	ns
**DCM Etiology**, **n **(%)		21 (32)	32 (48)	13 (20)	ns
**IHD Etiology**, **n **(%)		16 (24)	38 (58)	12 (18)	ns
**LV Ejection Fraction**, %	37.7 ± 7.6	38.2 ± 8.4	38.0 ± 7.1	36.4 ± 7.9	ns
**LVEF**<**40**% &**NYHA Class II**-**III**	35 (26.5)	8 (22.8)	18 (51.4)	9 (25.7)	ns
**Glucose **(**mg**/**dL**) ±**SD**	102.5 ± 23.4	95.5 ± 10.6	102.1 ± 25.3	114.2 ± 27.7	0.001
**Insulin **(**mU**/**mL**) ±**SD**	11.1 ± 7.3	9.5 ± 4.8	11.3 ± 8.3	12.9 ± 7.4	0.04
**HOMA**-**IR **±**SD**	2.9 ± 2.5	2.2 ± 1.3	2.3 ± 2.8	3.9 ± 2.8	0.005
**HOMA index**>**2**.**5**, **n **(%)	58 (43.9)	11 (30.0)	32 (45.7)	15 (60)	0.008
**Total Cholesterol **(**mg**/**dL**) ±**SD**	185.7 ± 41.3	179.4 ± 34.0	188.3 ± 44.3	187.8 ± 42.4	ns
**HDL**-**Cholesterol **(**mg**/**dL**) ±**SD**	42.3 ± 11.9	41.2 ± 10.9	42.2 ± 13.2	44.4 ± 10.1	ns
**LDL**-**Cholesterol **(**mg**/**dL**) ±**SD**	118.4 ± 36.0	116.8 ± 29.1	120.2 ± 36.5	115.9 ± 44.1	ns
**Triglycerides**(**mg**/**dL**) ±**SD**	123.8 ± 82.5	107.4 ± 54.2	127.7 ± 74.2	137.4 ± 127.3	ns

### Determinants of IR

At univariate analysis, including CV risk factors, LV functional parameters and eNOS T^−786^→C polymorphism, the presence of IR (as defined by a HOMA index > 2.5) was associated with history of hypertension, presence of a more severe LV dysfunction with HF and the eNOS genotype (Table 2). However, at multivariate logistic analysis (Table 2), the history of hypertension (p = 0.03) and the genotype remained the only independent predictors of IR (Table 2). In particular, the presence of TC genotype was associated with 2.1-fold (CI% 1.0-5.7, p < 0.05) higher risk of IR while the mutated genotype (CC) increased the risk to 3.3 times (CI% 1.1-10.1, p = 0.03) after correction for all other factors. Conversely, in the same population, the presence of severe LV dysfunction with HF was predicted at both univariate and multivariate analysis only by the HOMA index value (2.4 CI% 1.1-5.5, p = 0.03) (Table 3).

**Table 2:** **Predictors of HOMA**-**IR at univariate and multivariate analysis by logistic regression**

**Variables**	** Univariate Analysis**		** Multivariate Analysis**	
	**OR(CI 95%)**	**p**-**value**	**OR(CI 95%)**	**p**-**value**
**Age**	0.9 (0.9-1.0)	0.6	1.0 (0.9-1.0)	0.4
**Male sex**	1.7 (0.7-4.3)	0.2	2.7 (0.9-7.9)	0.07
**Smoking habit**, n (%)	0.7 (0.3-1.4)	0.3	_	_
**Hypercholesterolemia** n (%)	0.7 (0.4-1.5)	0.4	_	_
**History of Hypertension**, n (%)	1.9 (0.8-3.7)	0.07	2.0 (0.9-4.6)	0.09
**CAD Familiarity**, n (%)	0.6 (0.3-1.3)	0.2	_	_
**Obesity**, n (%)	0.9 (0.4-2.4)	0.9	_	_
**Non**-**ischemic Etiology**	1.8 (0.9-3.7)	0.08	1.9 (0.9-4.5)	0.1
**TC genotype**	2.1 (0.9-5.0)	0.08	2.4 (1.0-5.8)	0.06
**CC genotype**	3.6 (1.3-10.7)	0.01	3.9 (1.3-12.7)	0.02
**LV Ejection Fraction**, %	0.9 (0.9-1.0)	0.1	1.0 (0.9-1.0)	0.9
**LVEF**<**40**% &**NYHA Class II**-**III**	2.4 (1.1-5.4)	0.03	2.0 (0.7-5.8)	0.2

**Table 3:** Predictors of severe LV dysfunction with HF at univariate and multivariate analysis by logistic regression

**Variables**	** Univariate Analysis**		** Multivariate Analysis**	
	**OR(CI 95%)**	**p**-**value**	**OR(CI 95%)**	**p**-**value**
**Age**	1.0 (1.0-1.1)	0.1	1.0 (0.9-1.1)	0.1
**Male sex**	1.5 (0.5-4.3)	0.5	1.3 (0.4-3.9)	0.7
**Smoking habit**, n (%)	0.9 (0.4-2.0)	0.9	_	_
**Hypercholesterolemia** n (%)	0.9 (0.4-2.0)	0.9	_	_
**History of Hypertension**, n (%)	0.7 (0.3-1.6)	0.4	_	_
**CAD Familiarity**, n (%)	0.5 (0.2-1.2)	0.1	0.6 (0.3-1.5)	0.3
**Obesity**, n (%)	1.9 (0.7-5.1)	0.2	_	_
**TC genotype**	1.3 (0.5-3.4)	0.5	_	_
**CC genotype**	1.8 (0.6-6.5)	0.2	_	_
**HOMA index**	2.3 (1.0-5.2)	<0.05	2.4 (1.1-5.4)	0.03

## Discussion

This study provides evidence that the T^−786^→C polymorphism in the promoter region of eNOS gene is the major independent determinant of IR in patients with both ischemic and non- ischemic cardiomyopathy. Interestingly, among all classic cardiovascular risk factors, IR is the only one independently associated with the extent of LV dysfunction and the presence of HF in this population. Taken together, our results suggest that in either ischemic or non- ischemic cardiomyopathy, IR may occur preferentially in patients with a genetic predisposition to endothelial dysfunction (eNOS gene polymorphism) and may worsen the severity of the cardiac disease. Specifically, the mutual order among (genetically determined) endothelial dysfunction, IR and LV dysfunction was found independently of etiology of cardiac disease.

A link between insulin homeostasis and endothelial function has been long documented [6-8]. Endothelial function is modulated by insulin through the stimulatory effects of the hormone on NO production [6,8]. Nitric oxide is synthesized from L-arginine by a family of enzymes called NO synthases (NOSs). The constitutively expressed eNOS gene, which maps on the 7q35–36 chromosome, is mainly expressed in endothelial cells. It has been shown that eNOS activity is modulated by insulin through a series of phosphorylations triggered by the hormone binding with its receptor on the endothelial cell membrane. Specifically, the insulin signaling cascade leads to a specific phosphorylation of eNOS that increases the enzyme activity [8].

Nevertheless, some experimental data suggest that primary abnormalities of eNOS function may influence insulin homeostasis. In fact, eNOS knockout mice have been reported to be hypertensive and insulin resistant [9,15,16]. One elegant study in a mouse model of eNOS partial knockout showed that the partial deletion of the gene does not alter *per se* insulin sensitivity and blood pressure. However, when challenged with nutritional stress, partial eNOS deficiency facilitates the development of IR and arterial hypertension, providing further evidence for the importance of this gene in predisposing to glycometabolic and vascular abnormalities [17]. Anyway, data showing a clear, exact causal order between eNOS gene expression, hypertension and insulin resistance are unavailable. Likewise, the mechanisms by which the primitive endothelial alteration can affect glucose homeostasis and systemic blood pressure are not fully known.

In keeping with the above experimental findings, in a population of patients with ischemic and non-ischemic cardiac disease, we found a clear correlation between eNOS gene promoter polymorphism, the occurrence of an insulin-resistant phenotype and the presence of arterial systemic hypertension. Given the location of -786T>C in the promoter region of the eNOS gene, it may affect eNOS expression levels. Actually, lower eNOS mRNA and serum nitrite/nitrate levels have been found in individuals with the -786C variant [9], and reporter gene studies have supported this functional role [18,19].

One possible hypothesis linking the primitive impairment of eNOS function with insulin resistance states that endothelial dysfunction causing systemic NO depletion may affect systemic vascular tone, leading to a decrease in blood flow to the myocardium and muscles. This in turn may reduce myocardial and muscular glucose uptake. As a result, high glucose levels in the blood stimulate insulin secretion and in the long run may cause IR and diabetes. Therefore, a genetic variation that affects NO regulation may contribute to both alteration in vascular tone and to IR. Consistent with this hypothesis, few clinical studies have shown a significant association between that T^−786^→C polymorphism in the promoter region of the eNOS gene and IR in both non-diabetic subjects and Type 2 diabetic patients [11,12]. In this framework, our study confirms and extends previous results in patients without known diabetes but with systolic LV dysfunction.

An additional finding of the present study was that in our population IR, which occurred preferentially in subjects with eNOS gene polymorphism, was also an independent determinant of a more severe cardiac dysfunction with HF, irrespective of etiology. A recent study showed that the T^−786^→C promoter polymorphism was specifically associated with a significant reduction in eNOS mRNA expression in myocardial tissue obtained from failing human myocardium, while a different eNOS polymorphism G(894)-->T of exon 7 was not. The authors concluded that the reduced eNOS expression associated with the promoter gene polymorphism might be involved in the pathogenesis of cardiac failure [12]. Our findings did not discover a direct relationship between eNOS polymorphism and the severity of LV dysfunction; however, these conditions were linked by the presence of IR.

Insulin resistance, often manifested clinically through the feature of the metabolic syndrome or type 2 diabetes mellitus, has reached epidemic levels in many nations throughout the world [5,20]. Furthermore the presence of diabetes mellitus is more than 7 times as potent risk factor for mortality in the non-ischemic and ischemic cardiomyopathy population [21]. Certainly, the eNOS defective gene cannot be the only cause leading to insulin resistance and cardiovascular damage. Rather, our results suggest that this condition might create an individual substrate where the addition of other common factors (such as high fat diet or altered lipid profile) is poorly tolerated and enough to predispose to development of insulin resistance and more severe cardiac damage.

Our study findings should be interpreted bearing in mind some limitations. Firstly, we acknowledge that our sample size may temper statistical estimations in some categories. Secondly, genetic and acquired factors able to condition the presence and the extent of cardiac damage and the development of HF in different individuals are multiple and interactions are complex. Accordingly, adequate experimental models and large longitudinal clinical studies are needed to better elucidate the pathogenetic and prognostic relevance of these observations.

## Conclusion

Nevertheless, this study first shows a clear link between a genetic endothelial dysfunction and abnormalities in glycometabolic profile in patients with both ischemic and non-ischemic cardiomyopathy. Confirming the impact of a defective eNOS gene in the development of IR and HF will have major clinical relevance for identifying subjects at higher risk among patients with cardiac dysfunction.

## Competing interests

The authors declare that no relationship with industry exists.

## Authors’ contributions

CV participated in the design of the study, carried out the molecular genetic studies, performed the statistical analysis and drafted the manuscript. MGA participated in the design of the study and participated in its design and coordination and helped to draft the manuscript. RL helped to draft the manuscript. MGC carried out the molecular genetic studies. CC helped to draft the manuscript. ALA helped to draft the manuscript. DN conceived of the study, and participated in its design and coordination and helped to draft the manuscript. All authors read and approved the final manuscript.

## Pre-publication history

The pre-publication history for this paper can be accessed here:

http://www.biomedcentral.com/1471-2350/13/92/prepub
